# Exercise attenuates polyglutamine‐mediated neuromuscular degeneration in a mouse model of spinal and bulbar muscular atrophy

**DOI:** 10.1002/jcsm.13344

**Published:** 2023-11-08

**Authors:** Tomoki Hirunagi, Hideaki Nakatsuji, Kentaro Sahashi, Mikiyasu Yamamoto, Madoka Iida, Genki Tohnai, Naohide Kondo, Shinichiro Yamada, Ayuka Murakami, Seiya Noda, Hiroaki Adachi, Gen Sobue, Masahisa Katsuno

**Affiliations:** ^1^ Department of Neurology Nagoya University Graduate School of Medicine Nagoya Japan; ^2^ Aichi Medical University Nagakute Japan; ^3^ Department of Neurology National Hospital Organization Suzuka Hospital Suzuka Japan; ^4^ Department of Neurology University of Occupational and Environmental Health School of Medicine Kitakyushu Japan; ^5^ Department of Clinical Research Education Nagoya University Graduate School of Medicine Nagoya Japan

**Keywords:** AMPK, exercise, polyglutamine disease, spinal and bulbar muscular atrophy

## Abstract

**Background:**

Spinal and bulbar muscular atrophy (SBMA) is a hereditary neuromuscular disorder caused by the expansion of trinucleotide cytosine–adenine–guanine (CAG) repeats, which encodes a polyglutamine (polyQ) tract in the *androgen receptor* (*AR*) gene. Recent evidence suggests that, in addition to motor neuron degeneration, defective skeletal muscles are also the primary contributors to the pathogenesis in SBMA. While benefits of physical exercise have been suggested in SBMA, underlying mechanism remains elusive.

**Methods:**

We investigated the effect of running exercise in a transgenic mouse model of SBMA carrying human *AR* with 97 expanded CAGs (AR97Q). We assigned AR97Q mice to exercise and sedentary control groups, and mice in the exercise group received 1‐h forced running wheel (5 m/min) 5 days a week for 4 weeks during the early stage of the disease. Motor function (grip strength and rotarod performance) and survival of each group were analysed, and histopathological and biological features in skeletal muscles and motor neurons were evaluated.

**Results:**

AR97Q mice in the exercise group showed improvement in motor function (~40% and ~50% increase in grip strength and rotarod performance, respectively, *P* < 0.05) and survival (median survival 23.6 vs. 16.7 weeks, *P* < 0.05) with amelioration of neuronal and muscular histopathology (~1.4‐fold and ~2.8‐fold increase in motor neuron and muscle fibre size, respectively, *P* < 0.001) compared to those in the sedentary group. Nuclear accumulation of polyQ‐expanded AR in skeletal muscles and motor neurons was suppressed in the mice with exercise compared to the sedentary mice (~50% and ~30% reduction in 1C2‐positive cells in skeletal muscles and motor neurons, respectively, *P* < 0.05). We found that the exercise activated 5′‐adenosine monophosphate‐activated protein kinase (AMPK) signalling and inhibited mammalian target of rapamycin pathway that regulates protein synthesis in skeletal muscles of SBMA mice. Pharmacological activation of AMPK inhibited protein synthesis and reduced polyQ‐expanded AR proteins in C2C12 muscle cells.

**Conclusions:**

Our findings suggest the therapeutic potential of exercise‐induced effect via AMPK activation in SBMA.

## Introduction

Spinal and bulbar muscular atrophy (SBMA) is an inherited neurological disorder characterized by slowly progressive weakness and atrophy of limb and bulbar muscles, which exclusively affects adult males.^1^ SBMA is caused by the expansion of trinucleotide cytosine–adenine–guanine (CAG) repeats, which encodes a polyglutamine (polyQ) tract in the *androgen receptor* (*AR*) gene.^2^ PolyQ‐expanded AR translocates and accumulates in the nucleus by binding to AR ligands, such as testosterone, which leads to cellular damage and neuronal and muscular degeneration via several mechanisms, including transcriptional dysregulation, axonal transport disruption, impairment of protein degradation and mitochondrial dysfunction.^3^, ^4^

A neuropathological hallmark of SBMA is degeneration of lower motor neurons accompanied by nuclear accumulation of polyQ‐expanded AR protein, with muscle histopathology showing both neurogenic and myopathic changes.^5^, ^6^ Recent evidence suggests that not only motor neurons but also skeletal muscles are the primary contributors of disease pathogenesis in SBMA. Defective excitation–contraction coupling and mitochondrial respiration emerge prior to the onset of motor dysfunction in mouse models.^7^ Muscle‐specific overexpression of wild‐type (WT) *AR* in mice causes SBMA‐like neuromuscular disease.^8^ Overexpression of insulin‐like growth factor 1 (IGF‐1) in skeletal muscle,^9^ muscle‐specific excision of mutant *AR* ^10^ or peripheral *AR* gene suppression^11^ ameliorates neuromuscular degeneration in mouse models of SBMA. These observations support the hypothesis that skeletal muscle is a primary target of polyQ‐expanded AR toxicity.

Physical exercise has attracted attention because of its beneficial effects on neuromuscular disorders. Exercise is beneficial in both neurodegenerative disorders, such as Alzheimer's disease, Parkinson's disease and amyotrophic lateral sclerosis, and myopathies, such as Duchenne muscular dystrophy and myotonic dystrophy.^12^ ^,^ ^S1^ To date, there are three clinical studies of physical exercise in SBMA patients. The first study reported that aerobic training was of little benefit and not well tolerated.^13^ The second study reported that some patients respond to functional exercise,^14^ and the latest study showed potential benefits of high‐intensity exercise in SBMA.^15^ However, the underlying mechanism of the effect of physical exercise on SBMA pathology largely remains to be investigated.

Previous studies have addressed the effects of exercise on polyQ‐mediated neuronal and muscular toxicities. Several studies showed that running exercise improves disease phenotype in mouse models of Huntington's disease,^16^, ^17^, ^18^, ^19^ but others showed that exercise exacerbates disease symptoms.^20^, ^21^ The discrepancy between these results reflects the fact that the effect of exercise depends on its type, timing, duration and intensity. Interestingly, mild exercise during an early period showed long‐term effects on survival and motor function in a mouse model of spinocerebellar ataxia type 1.^22^ The effect of exercise was also investigated in an AR113Q knock‐in mouse model of SBMA, which showed that treadmill exercise did not show beneficial effect on survival and muscle fibre sizes of the mice.^23^ Instead, about 30% of the mice died without completing the exercise, which raised the concern that the timing of exercise might have been too late to ameliorate the disease phenotype.

In this study, we investigated the effect of exercise in our AR97Q transgenic mouse model of SBMA and found that aerobic exercise during the early stage of the disease showed improvement in motor function and survival with amelioration of neuronal and muscular histopathology. Furthermore, based on the findings that the aerobic exercise activated 5′‐adenosine monophosphate‐activated protein kinase (AMPK) signalling pathway in the skeletal muscles of AR97Q mice, we explored the therapeutic potential of AMPK activation in a muscle cell model of SBMA.

## Methods

### Animals and study design

This study was approved by the Animal Experiment Committee of Nagoya University Graduate School of Medicine. The transgenic mouse model of SBMA carrying human *AR* with 97 expanded CAGs (AR97Q) was generated and maintained on a C57BL/6J background in the animal facility as previously described.^24^ Male AR97Q mice were used in this study because they show progressive muscular atrophy and weakness as well as an SBMA‐like pathology. Male AR97Q mice exhibit motor deficits with the onset at 8–9 weeks of age, followed by weight loss and impairment of movement in the advanced stage (after 12–13 weeks of age).^24^ This study was designed to evaluate the effect of exercise during the early period on the later‐stage disease phenotype of AR97Q mice. Therefore, 4‐week exercise was performed during the presymptomatic period (from 5 to 9 weeks of age). Survival, body weight and motor function were analysed throughout the entire period (*Figure* *S1A*). Littermates were used for the phenotypic analysis to standardize the conditions of breeding and upbringing as much as possible. Mouse protocols were used in accordance with the National Institutes of Health Guide for the Care and Use of Laboratory Animals.

### Exercise protocol

Mice in the exercise group were trained to run on a forced running wheel system (FRW800, Lafayette Instrument, IN). According to a previous report,^S2^ 3, 6 and 12.6 m/min correspond to 65%, 70% and 80% of the maximal oxygen consumption of mice, respectively. Preliminary loading of high‐intensity exercise (10 m/min) to AR97Q mice was attempted, but it did not achieve the expected intensity because the mice often did not run onto the wheel (data not shown). Therefore, a low‐ to moderate‐intensity exercise regimen (5 m/min) was selected for the present study. Mice were acclimated to the procedure for 1 week at 5 m/min for 30 min/day, 3 days/week, and were then subjected to exercise at 5 m/min for 60 min/day, 5 days/week. Due to the disease progression in AR97Q mice,^24^ some of the mice beyond 10 weeks of age were found unable to continue exercise because of severe muscle weakness and/or a moribund condition. All mice were able to perform the aerobic exercise at 5 m/min for 1 h without interruption until 9 weeks of age, but ~10% and ~30% of mice were unable to complete exercise at 10 and 12 weeks of age, respectively (data not shown). Therefore, exercise was started at 5 weeks and terminated at 9 weeks of age in all mice. This aerobic exercise regimen did not significantly increase mRNA levels of *citrate synthase* (*CS*), a well‐known biochemical indicator of adaptation to exercise,^S3^ in the quadriceps of WT mice at 9 weeks of age. In contrast, the same exercise regimen increased expression of *CS* in the quadriceps of AR97Q mice (~2‐fold increase, *P* < 0.05) (*Figure* *S1B*). All exercise in the regimen was continuously monitored. When tissue samples were collected at 9 weeks of age, mice were euthanized the day after their last bout of the exercise.

### Behavioural analysis

All the tests were performed on a weekly basis by examiners who were blinded to the details of the experiment. Rotarod performance was assessed weekly using an Economex Rotarod (Ugo Basile, Comerio, Italy), as previously described.^25^ The grip strength was measured with a Grip Strength Meter (MK‐380M, Muromachi Kikai, Tokyo, Japan). To impute values of mice after death, the final body weights of all mice were carried forward when calculating group mean values as described in previous reports.^S4^ ^,^ ^S5^ The values of grip strength and rotarod performance after death were imputed to zero. The footprint analysis was performed as described elsewhere.^26^

### Immunoblotting

Equal amounts of protein were separated on sodium dodecyl sulfate–polyacrylamide gel electrophoresis (SDS–PAGE) gels and transferred to Hybond P membranes (GE Healthcare, Piscataway, NJ) as previously described.^27^ Details are described in the Methods section in the supporting information.

### Histology and immunohistochemistry

Anaesthetized mice were perfused with a 4% paraformaldehyde fixative in phosphate buffer at pH 7.4. Tissues were dissected and processed for histological and immunohistochemical analysis. Details are described in the Methods section in the supporting information.

### Quantitative analysis of immunohistochemistry

1C2‐positive cells from thoracic spinal cord and quadriceps muscles were assessed as previously described.^28^ Details are described in the Methods section in the supporting information.

### Quantification of mRNA levels

The mRNA levels were quantified by real‐time PCR. Details are described in the Methods section in the supporting information.

### Serum testosterone assay

Serum testosterone was assayed using the Testosterone EIA Kit (No. 582701, Cayman Chemical, MI) according to the manufacturer's instructions.

### Cell culture

C2C12‐AR97Q cells were established as previously described.^29^ The cells were differentiated in Dulbecco's modified Eagle's medium (Life Technologies, Carlsbad, CA) with 2% foetal horse serum and 10 nM of dihydrotestosterone for 48 and 24 h before experiments, respectively. After differentiation, 5‐aminoimidazole‐4‐carboxamide‐1‐β‐d‐ribofuranoside (AICAR) (Fujifilm) was dissolved in the medium at a concentration of 0.5 mM for the indicated hours. For the interventions, the cells were treated of AICAR with 1 μM of MG132 (Sigma‐Aldrich) or 100 nM of bafilomycin A1 (Sigma‐Aldrich) for 24 h in accordance with a previous report.^30^ Mitochondria were labelled by 100 nM of MitoTracker Green FM (Invitrogen) and visualized under a fluorescent microscope (BZ‐X710, Keyence). Autophagy flux assay was performed with or without treatment of 50 μM of chloroquine diphosphate (Sigma‐Aldrich) for 6 h. Protein turnover assay was performed using 1 mg/mL of cycloheximide (CHX) (Sigma‐Aldrich) for the indicated hours.

### Microarray analysis

Gene expression in the quadriceps was assessed using SurePrint G3 Mouse GE v2 8x60K Microarray (Agilent Technologies, Santa Clara, CA). Details are described in the Methods section in the supporting information.

### Statistical analysis

The data obtained from animal experiments were analysed using unpaired *t* tests for two‐group comparisons (sedentary and exercise groups) and two‐factor analysis of variance (ANOVA) with Tukey's post hoc tests for four‐group comparisons (sedentary and exercise groups in WT and AR97Q mice). The data obtained from cellular experiments were analysed using unpaired *t* tests for two‐group comparisons and one‐way ANOVA with Tukey's post hoc tests for multiple comparisons, unless otherwise noted. The survival rate was analysed using the Kaplan–Meier and log‐rank tests. In all cases, *P* values of <0.05 were considered to be statistically significant. Statistical analyses were performed using SPSS Version 23.0 (SPSS Japan, Tokyo, Japan) or GraphPad Prism 8 (GraphPad Software Inc., San Diego, CA). Error bars indicate the standard deviation unless otherwise noted.

### Data availability

The data of microarray analysis have been submitted to the National Center for Biotechnology Information Gene Expression Omnibus (https://www.ncbi.nlm.nih.gov/geo/), and the accession number of the submission is GSE209885.

## Results

### Exercise during the early stage ameliorated motor dysfunction and extended the life span of AR97Q mice

**Figure 1:**
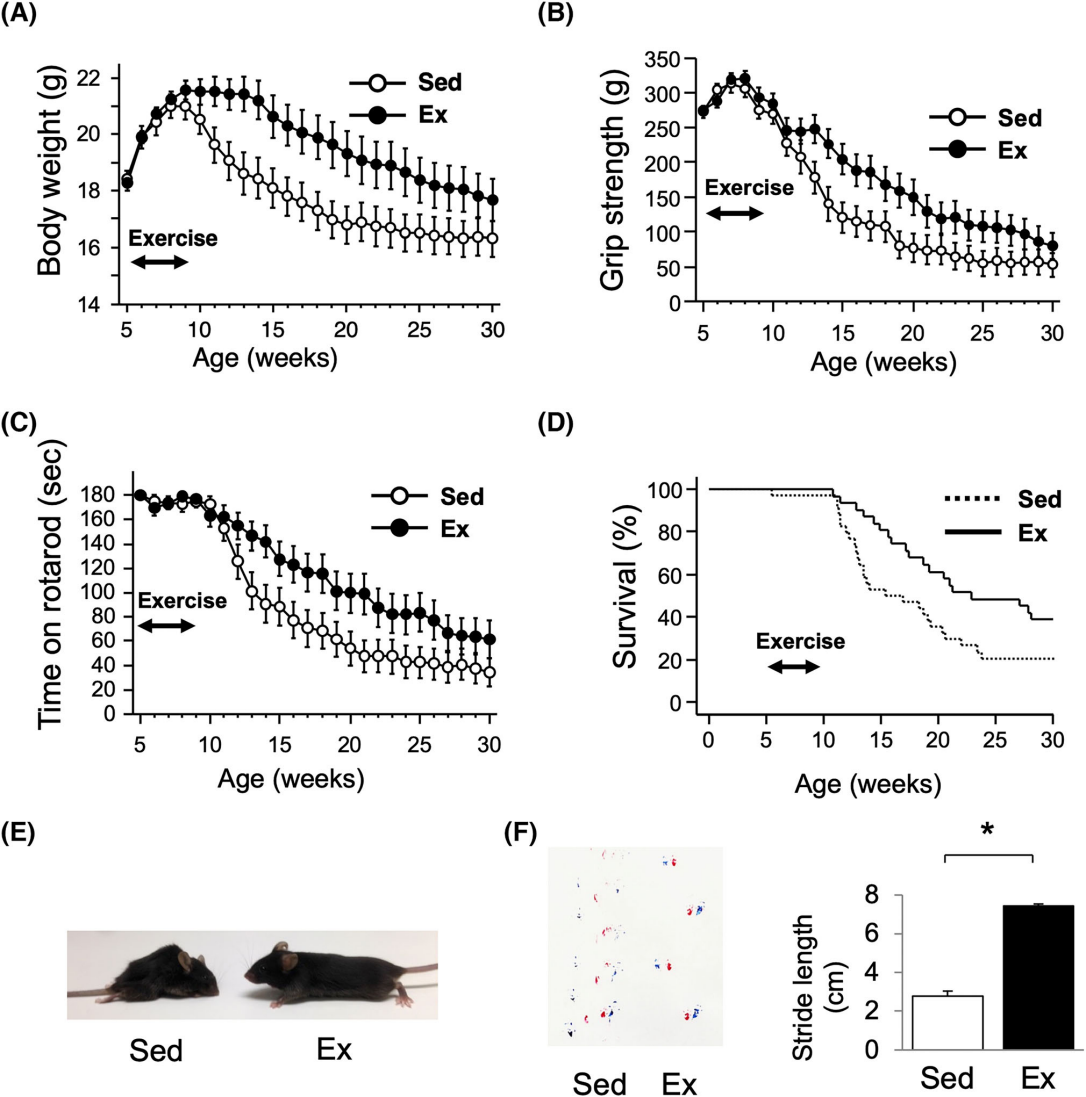
Exercise ameliorates motor dysfunction and extends the life span of AR97Q mice. Exercise was started from a presymptomatic stage (5 weeks of age) and maintained for 4 weeks. (A) Body weight, (B) grip strength, (C) rotarod performance and (D) survival rate of AR97Q mice in the sedentary (Sed) (*n* = 34) and exercise (Ex) (*n* = 31) groups are shown. To impute values of mice after death, the final body weights of all mice were carried forward when calculating group mean values. The values of grip strength and rotarod performance after death were imputed to zero. Error bars indicate the standard error of the mean. Body weight, grip strength, rotarod performance and survival were improved by the exercise (*P* < 0.05 at 13 weeks by two‐tailed *t* tests for body weight, grip strength, rotarod performance and *P* < 0.05 by log‐rank test for survival rate). (E) Representative photograph and (F) footprints of AR97Q mice in the Sed and Ex groups at 13 weeks of age. Front and hind paws are shown in red and blue, respectively. The stride length of the mice is quantified (*n* = 6 per group). *P < 0.05 by unpaired *t* test.

A low‐ to moderate‐intensity (5 m/min) exercise regimen (started at 5 weeks, for 4 weeks) was selected for the present study (*Figure* *S1A*). The aerobic exercise did not affect the motor function, weight gain and survival in WT mice (*Figure* *S2*). In AR97Q mice, the exercise significantly attenuated body weight loss at 13 weeks of age (*P* < 0.05) (*Figure* *1* *A*) and retained motor function, measured with grip strength (~40% increase at 13 weeks of age, *P* < 0.05) (*Figure* *1* *B*) and rotarod performance test (~50% increase at 13 weeks of age, *P* < 0.05) (*Figure* *1* *C*), and extended the life span (median survival 23.6 vs. 16.7 weeks, *P* < 0.05) (*Figure* *1* *D*) compared to those of the sedentary mice. The exercise also mitigated muscle atrophy (*Figure* *1* *E*) and increased stride length (~2.7‐fold increase at 13 weeks of age, *P* < 0.001) (*Figure* *1* *F*) in AR97Q mice.

### Exercise ameliorated histopathological findings in AR97Q mice at 13 weeks of age

**Figure 2:**
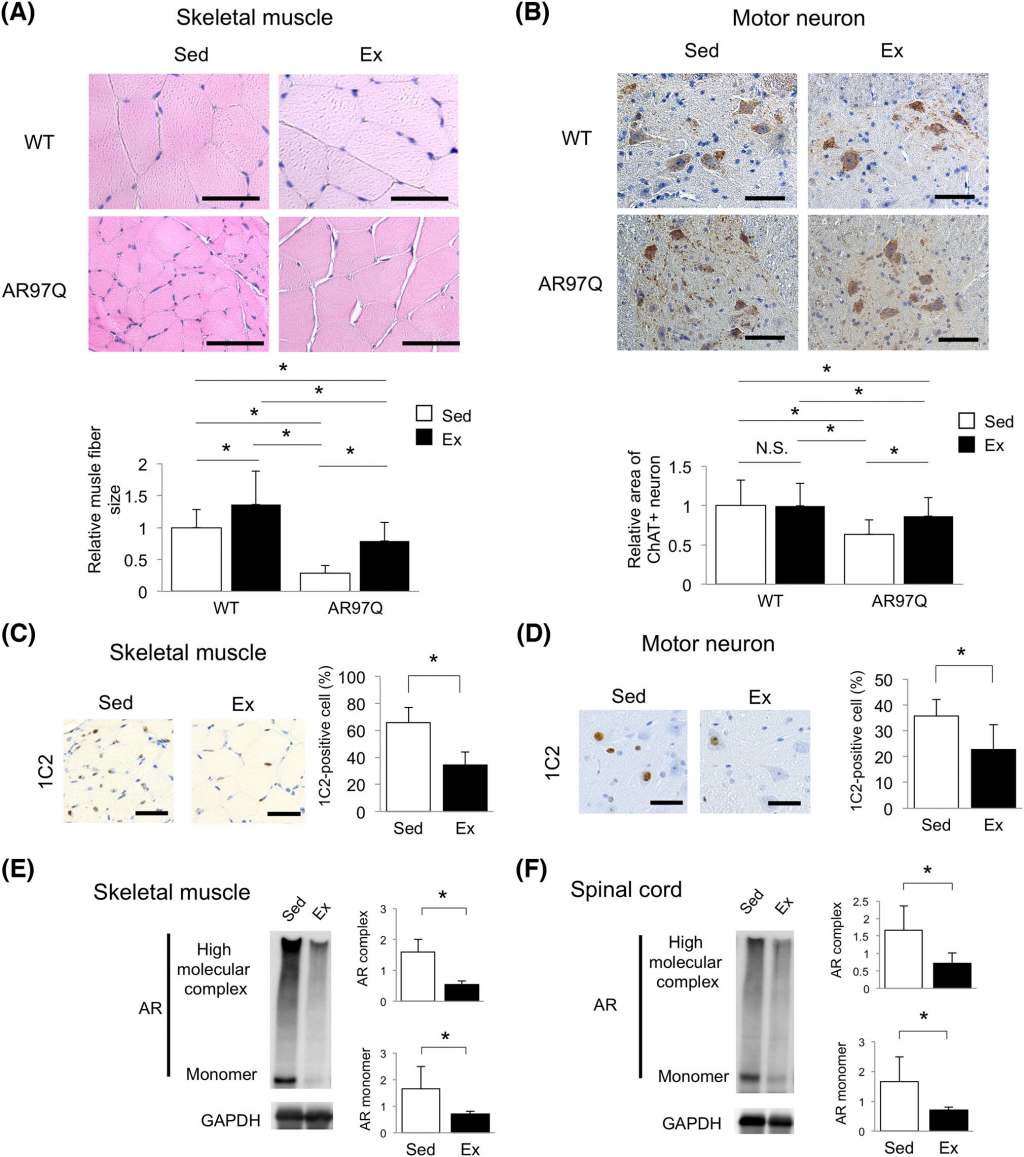
Exercise ameliorates histopathological findings in AR97Q mice at 13 weeks of age. (A) Haematoxylin and eosin (HE) staining of the quadriceps and quantification of relative muscle fibre size at 13 weeks (*n* = 3 per group, ≥30 fibres per mouse). (B) Anti‐choline acetyltransferase (ChAT) immunostaining of spinal motor neurons and quantification of relative area of ChAT‐positive (+) neurons (*n* = 3 per group, ≥5 axial sections). Immunostaining with 1C2 antibody against expanded polyQ proteins and quantification of 1C2‐positive cells in (C) skeletal muscles and (D) motor neurons of AR97Q mice (*n* = 6 per group). Immunoblots for AR of (E) the quadriceps and (F) spinal cords of AR97Q mice (*n* = 4–5 per group). Ex, exercise; Sed, sedentary. Scale bar = 50 μm. *P < 0.05 by (A, B) two‐factor ANOVA with Tukey's post hoc tests or (C–F) unpaired *t* test.

We examined histopathological findings in AR97Q and WT mice in the exercise group at 13 weeks of age, at which half of the sedentary AR97Q mice had died. Haematoxylin and eosin staining of skeletal muscles showed that muscle fibre sizes were retained in the quadriceps of exercised AR97Q mice (~2.8‐fold increase in fibre size, *P* < 0.001), contrasting with smaller sizes in the sedentary mice (*Figure* *2* *A*). The exercise also increased muscle fibre sizes in WT mice by ~1.3‐fold (*P* < 0.001), but the effect was modest compared to AR97Q mice (*Figure* *2* *A*). Immunohistochemistry for choline acetyltransferase (ChAT), a histological marker of motor neurons, showed that the exercise did not affect motor neuron sizes in WT mice, whereas it attenuated motor neuron shrinkage in AR97Q mice (~1.4‐fold increase in neuron size, *P* < 0.001) (*Figure* *2* *B*). Immunostaining with 1C2 antibody against polyQ proteins showed that the exercise attenuated nuclear accumulation of polyQ‐expanded AR in skeletal muscles (~50% reduction in 1C2‐positive fibre, *P* < 0.05) (*Figure* *2* *C*) and motor neurons (~30% reduction in 1C2‐positive neurons, *P* < 0.05) (*Figure* *2* *D*). Correspondingly, immunoblots showed decreased protein levels of high molecular weight complex and monomeric AR in both the skeletal muscles and the spinal cord (*Figure* *2* *E*,*F*).

### Exercise attenuated polyglutamine‐expanded AR accumulation in skeletal muscles at 9 weeks of age

**Figure 3:**
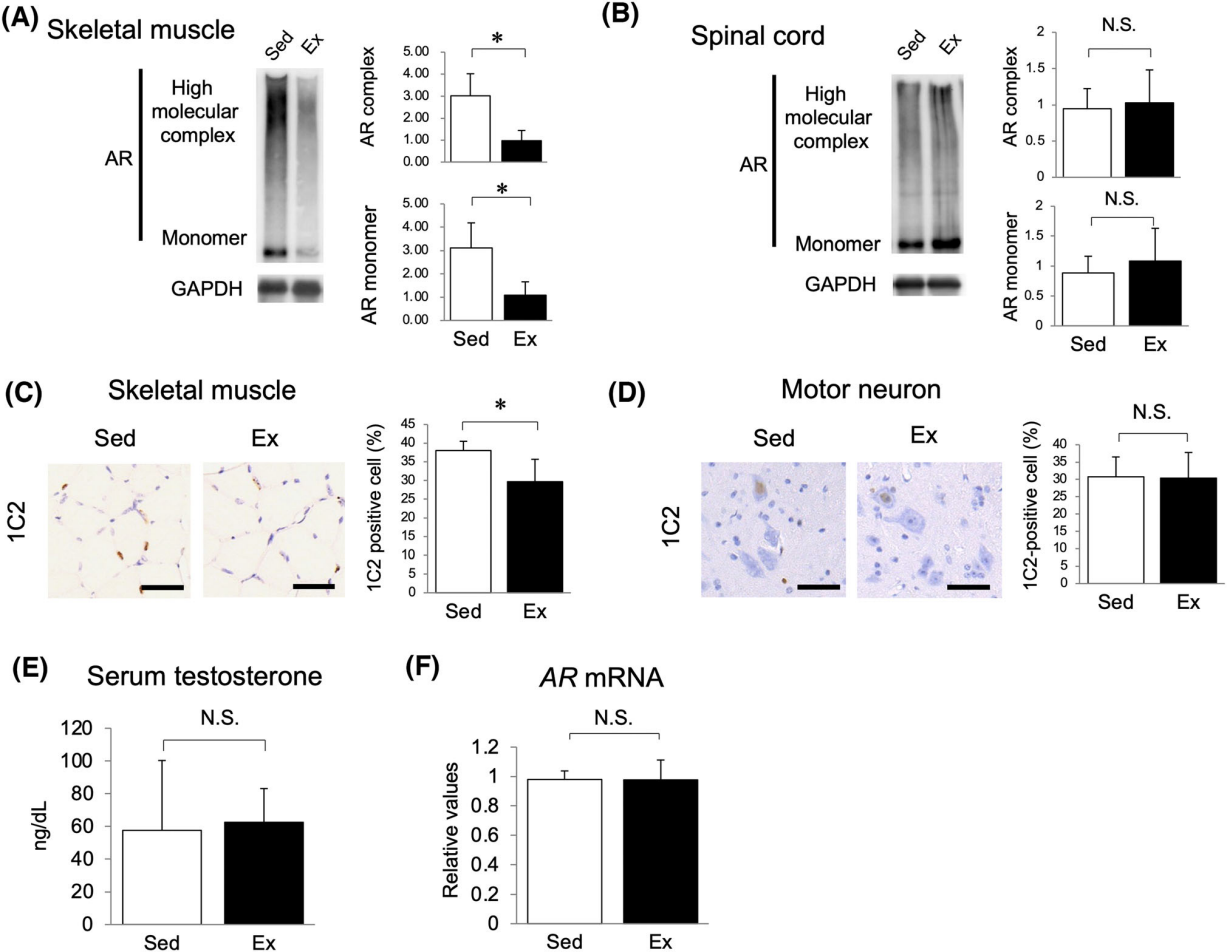
Exercise attenuated polyglutamine‐expanded AR accumulation in skeletal muscles at 9 weeks of age. Immunoblots for AR of (A) the quadriceps and (B) spinal cord of AR97Q mice (*n* = 3 per group). Immunostaining with 1C2 antibody against expanded polyQ proteins and quantification of 1C2‐positive cells in (C) skeletal muscles and (D) motor neurons of AR97Q mice (*n* = 5–6 per group). (E) Serum testosterone levels of AR97Q mice at 9 weeks of age (*n* = 3 per group). (F) Mutant *AR* mRNA levels in the quadriceps of AR97Q mice at 9 weeks of age (*n* = 3 per group). Ex, exercise; Sed, sedentary. Scale bar = 50 μm. Error bars indicate SEM. *P < 0.05 by unpaired *t* test.

To investigate the primary effects of early aerobic exercise, we evaluated polyQ‐expanded AR accumulation at 9 weeks of age, at the timing of termination of the exercise. Surprisingly, immunoblots for AR showed marked reduction of high molecular weight complex and monomeric AR in skeletal muscles of the exercised mice (*Figure* *3* *A*), whereas there was no change of those in the spinal cord (*Figure* *3* *B*). Correspondingly, immunostaining with 1C2 antibody showed reduced number of immunopositive cells in skeletal muscles (~25% reduction, *P* < 0.05) (*Figure* *3* *C*), whereas no differences of those were observed in motor neurons (*Figure* *3* *D*). Serum testosterone (*Figure* *3* *E*) and mutant *AR* gene expression (*Figure* *3* *F*) were almost at the same level between the sedentary and early exercise groups at 9 weeks of age.

### Exercise attenuated fast‐ to slow‐twitch muscle fibre‐type switching in AR97Q mice

**Figure 4:**
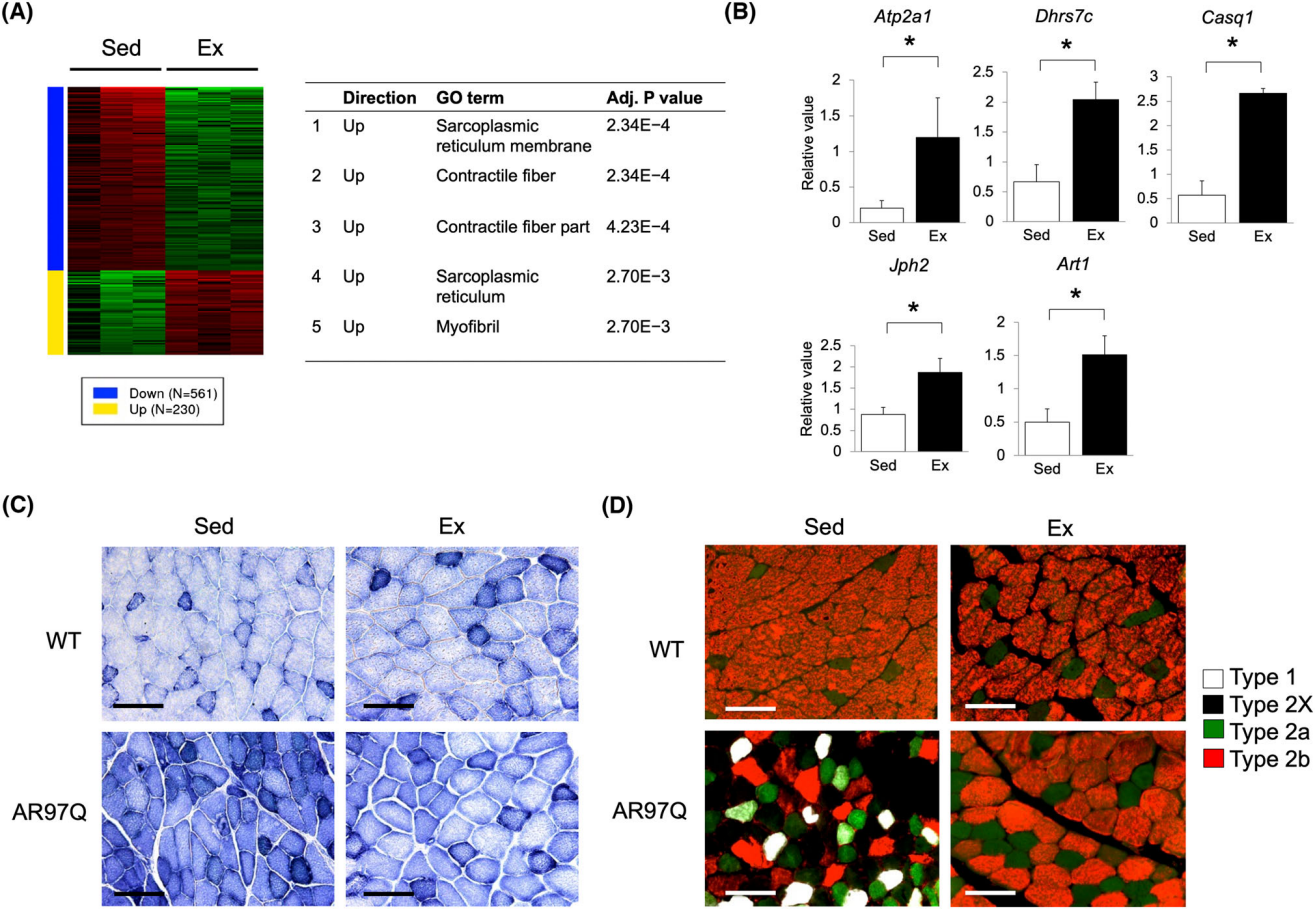
Exercise attenuates fast‐ to slow‐twitch muscle fibre‐type switching in AR97Q mice. (A) Heatmap and the top 5 GO terms (cellular component) of differentially expressed genes (FDR < 0.05, absolute log FC ≥ 1) from microarray data of the quadriceps of AR97Q mice between the sedentary (Sed) and exercise (Ex) groups (*n* = 3 per group). (B) Validation of genes of sarcoplasmic reticulum membrane by quantitative PCR (*n* = 3 per group). (C) NADH staining of the quadriceps of WT and AR97Q mice at 9 weeks of age. (D) Immunofluorescence of type 1 (white), type 2a (green) and type 2b (red) myosin heavy chain‐positive fibres (bottom) of the quadriceps of WT and AR97Q mice at 9 weeks of age. Muscle fibres not stained (black) indicate type 2X. Scale bar = 100 μm. *P < 0.05 by unpaired *t* test.

To investigate the molecular pathway underlying the effect of exercise, we performed microarray gene expression analysis of the quadriceps of AR97Q mice at 9 weeks of age with or without the early exercise. Principal component analysis showed separation of gene expression between the sedentary and early exercise groups (*Figure* *S3A*). Genes with false discovery rate (FDR) <0.05 and absolute log fold change (FC) ≥1 were defined as differentially expressed genes (DEGs), which included 230 upregulated and 561 downregulated genes (*Figure* *4* *A*). By Gene Ontology analysis (cellular component), we identified the five enriched categories (adj. *P* value <0.01) in the upregulated DEGs (*Figure* *4* *A*). No significant categories were enriched in the downregulated DEGs.

To validate the results of microarray analysis, we confirmed upregulation of the five genes in the category of sarcoplasmic reticulum membrane by quantitative PCR analysis (*Figure* *4* *B*). Interestingly, the top 5 categories of the upregulated DEGs include many genes that specifically expressed in fast‐twitch skeletal muscles (*Figure* *S3B*). Fast‐ to slow‐twitch muscle fibre‐type switching was reported to be an early feature in SBMA muscles.^31^, ^32^ Therefore, we investigated muscle fibre types of the quadriceps of AR97Q mice in the sedentary and early aerobic exercise groups using nicotinamide adenine dinucleotide (NADH) staining (*Figure* *4* *C*) and immunofluorescence for myosin heavy chain (MHC) type 1, type 2a and type 2b (*Figure* *4* *D*). As expected, both the NADH staining and the MHC staining showed that the quadriceps of AR97Q mice in the early exercise group contained more fast‐twitch muscle fibres compared to those in the sedentary group at 9 weeks of age. In WT mice, there were no apparent differences of muscle fibre types of quadriceps muscles between the sedentary and exercise groups (*Figure* *4* *C*,*D*). These results suggest that the early aerobic exercise attenuated fast‐ to slow‐twitch muscle fibre‐type switching in AR97Q mice.

We also analysed the top 30 DEGs in the microarray results (listed in *Figure* *S4*). Downregulated DEGs include denervation markers of skeletal muscles, such as *Chrng* and *Myh3*, which are reported to be elevated in skeletal muscles of SBMA mouse models,^9^, ^31^ suggesting that the early aerobic exercise attenuated muscle denervation in AR97Q mice.

### Exercise induced mitochondria biogenesis and inhibited protein synthesis pathway via AMPK signal activation in skeletal muscles of AR97Q mice

**Figure 5:**
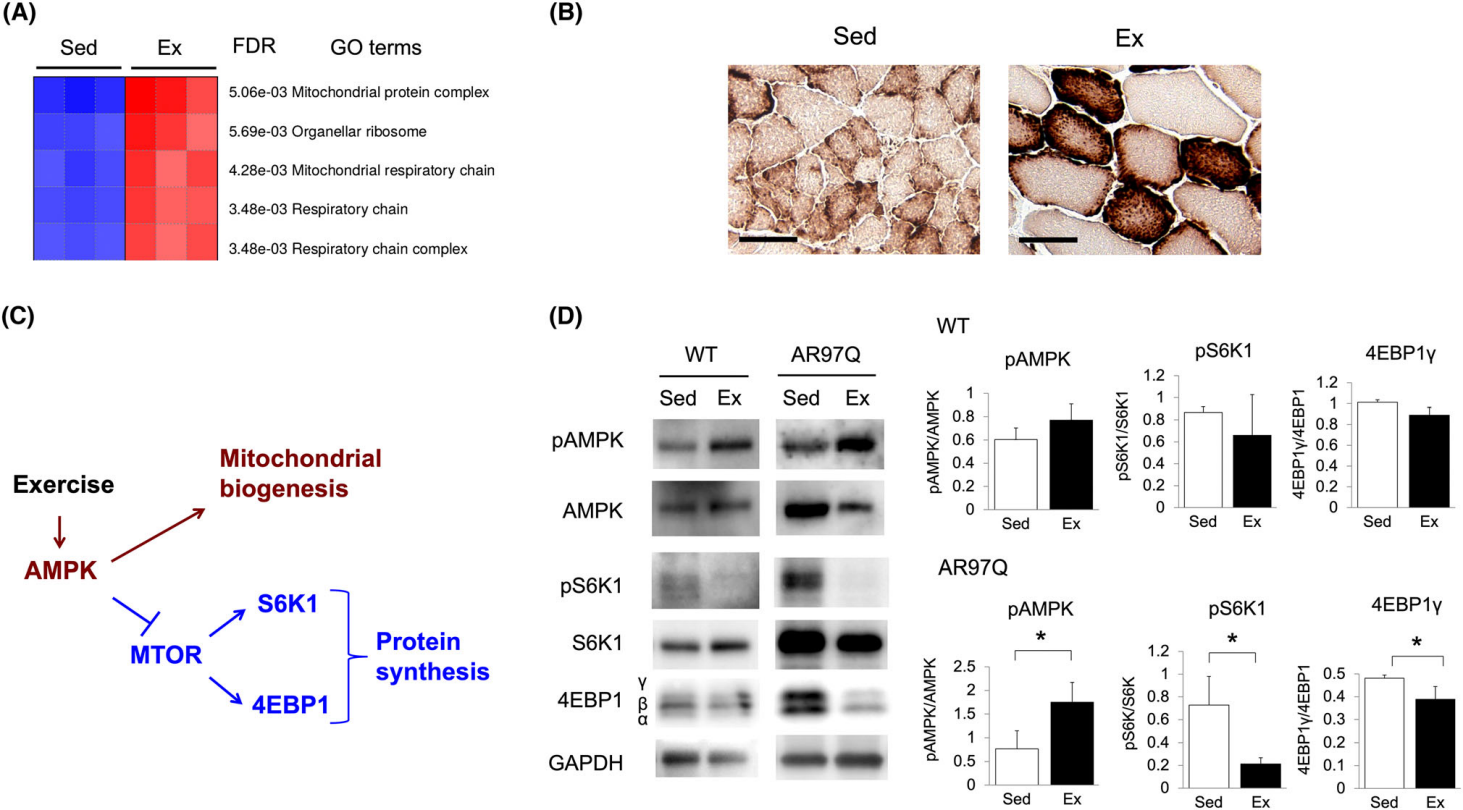
Exercise activates AMPK signalling in skeletal muscles of AR97Q mice. (A) Parametric Gene Set Enrichment Analysis (GO terms: cellular component) shows upregulation of genes of mitochondrial protein complex (*n* = 3 per group). (B) Mitochondrial cytochrome *c* oxidase staining of the quadriceps of AR97Q mice at 9 weeks of age. Scale bar = 100 μm. (C) Schematic of AMPK signalling pathways, which regulate mitochondrial biogenesis and protein synthesis in skeletal muscles (stimulatory and inhibitory in red and blue, respectively). (D) Immunoblots for phosphorylation (Thr‐172), total AMPK, phosphorylation (Thr‐389), total S6K1 and 4EBP1 of the quadriceps of WT and AR97Q mice at 9 weeks of age (*n* = 3 per group). Ex, exercise; FDR, false discovery rate; Sed, sedentary. *P < 0.05 by unpaired *t* test.

We further performed an exploratory parametric analysis of microarray gene expression data (Parametric Gene Set Enrichment Analysis [PGSEA]) to determine DEG clusters rather than individual genes.^S6^ PGSEA showed upregulation of genes of mitochondrial protein complex in skeletal muscles of AR97Q mice in the early exercise group (*Figure* *5* *A*). Correspondingly, mitochondrial cytochrome *c* oxidase staining showed preservation of the stained fibres in the exercised mice and diminished staining in the sedentary mice (*Figure* *5* *B*). As mitochondrial biogenesis is induced by exercise through activation of AMPK signalling in murine and human muscles,^S7^ ^,^ ^S8^ we focused on AMPK signalling in skeletal muscles of AR97Q mice. Kyoto Encyclopedia of Genes and Genomes (KEGG) pathway analysis^S9^ ^,^ ^S10^ of AMPK signalling using microarray results (*Figure* *S5*) showed upregulation of *glucose transporter type 4* (*Glut4*), which is known to be upregulated by exercise in parallel with mitochondrial biogenesis.^S11^ Interestingly, we also found downregulation of mammalian target of rapamycin (mTOR) pathway that regulates protein synthesis (*Figure* *S5*), which was previously shown to be elevated in SBMA muscles.^31^, ^33^ We hypothesized that exercise‐induced AMPK signalling activation facilitated mitochondrial biogenesis and inhibited mTOR signalling in skeletal muscles of AR97Q mice (*Figure* *5* *C*). To determine whether AMPK signalling was activated by the exercise, we examined phosphorylation levels of AMPK in skeletal muscles. Consistent with our hypothesis, immunoblots for phosphorylation (Thr‐172) and total AMPK showed increased phosphorylated levels of AMPK by 2.3‐fold (*P* < 0.05) in the quadriceps of AR97Q mice in the exercise group (*Figure* *5* *D*). Correspondingly, phosphorylation levels of ribosomal protein S6 kinase B1 (S6K1) (~70% decrease, *P* < 0.05) and eukaryotic translation initiation factor 4E binding protein 1 (4EBP1) (~20% decrease, *P* < 0.05), which are responsible for protein synthesis in mTOR signalling,^S12^ were decreased by the exercise (~70% and ~20% reduction in S6K1 and 4EBP1, respectively, *P* < 0.05) (*Figure* *5* *D*). In WT mice, the exercise similarly tended to increase phosphorylation levels of AMPK and decrease phosphorylation levels of S6K1 and 4EBP1 in skeletal muscles, although these effects were modest and not statistically significant (*Figure* *5* *D*).

### Autophagy, IGF‐1/Akt signalling and BDNF expression were not elevated by exercise in skeletal muscles of AR97Q mice

Given that both AMPK activation and mTOR inhibition induce autophagy,^S13^ we hypothesized that the exercise reduced polyQ‐expanded AR accumulation through activation of autophagy. In contrast to our expectation, many of autophagy‐related genes were downregulated in the quadriceps of AR97Q mice with the exercise (*Figure* *S6A*). Correspondingly, we found that the exercise reduced levels of key autophagy proteins,^S14^ beclin1 (Becn1) by ~50% (*P* < 0.05) and microtubule‐associated protein light‐chain 3 isoform II (LC3‐II) by ~60% (*P* < 0.05) (*Figure* *S6B*). AMPK signalling can be associated with cytokines and other peptides expressed in skeletal muscles, called myokines.^S15^ ^,^ ^S16^ Here, we focused on two exercise‐induced myokines: IGF‐1 and brain‐derived neurotrophic factor (BDNF). A previous study showed that muscle expression of human IGF‐1 facilitated AR clearance via the ubiquitin–proteasome system through phosphorylation of AR by Akt.^9^ Muscle expression of BDNF was also shown to improve clinical phenotype of AR97Q mice.^34^ However, expression of *Igf1* and *Bdnf* (*Figure* *S6C*) and phosphorylation levels of Akt (*Figure* *S6D*) in the quadriceps of AR97Q mice did not differ between the sedentary and exercise groups. These results indicate that phenotypic amelioration of AR97Q mice by the early exercise is unlikely because of autophagy, IGF‐1/Akt signalling or BDNF elevation in skeletal muscles.

### Pharmacological activation of AMPK reduced AR aggregation by inhibiting protein synthesis pathway in C2C12 muscle cells

**Figure 6:**
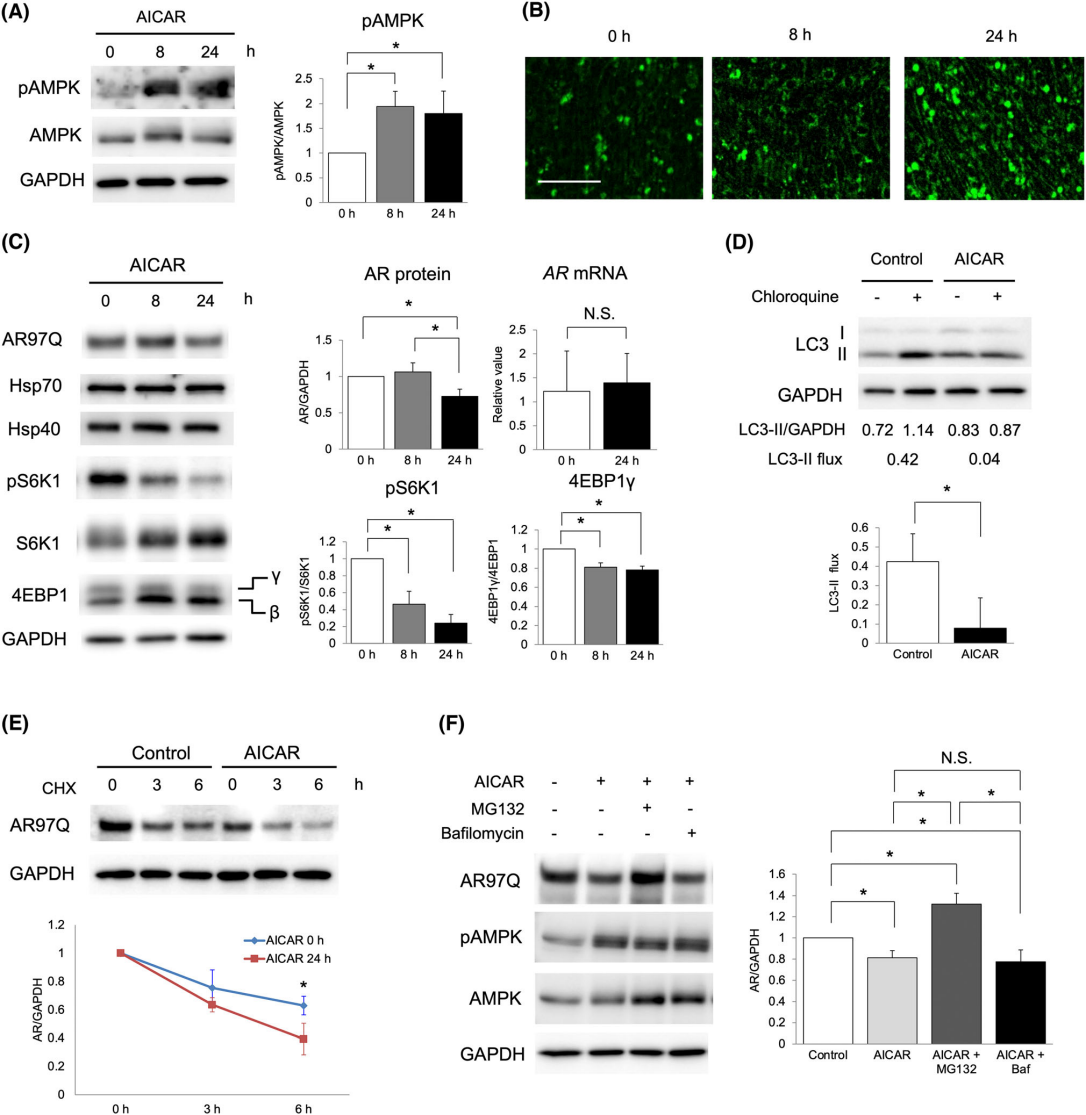
AMPK activation by AICAR reduces polyQ‐expanded AR by inhibiting protein synthesis pathway in C2C12 cells. (A) Immunoblots show phosphorylation (Thr‐172) and total AMPK levels in C2C12 cells stably expressing *AR* with 97 CAG repeats (C2C12‐AR97Q) treated with 0.5‐mM AICAR for 0, 8 or 24 h. (B) Visualization of mitochondria in C2C12‐AR97Q cells treated with 0.5‐mM AICAR for 0, 8 and 24 h. Scale bar = 100 μm. (C) Immunoblots for AR, phosphorylation (Thr‐389) and total S6K1, and 4EBP1 in C2C12‐AR97Q cells treated with 0.5‐mM AICAR for 0, 8 and 24 h. Graphs show densitometry quantification of relative AR protein, pS6K1, 4EBP1γ levels (*n* = 3) and *AR* mRNA levels analysed by quantitative PCR (*n* = 4). (D) C2C12‐AR97Q cells treated with 0.5‐mM AICAR for 0 (control) or 24 h are immunoblotted for LC3‐II in the presence or absence of 50‐μM chloroquine for 6 h. The ratio of LC3‐II to GAPDH is determined by densitometry quantification. The graph shows the LC3‐II flux calculated as the difference between the ratio of LC3‐II to GAPDH in the presence or absence of chloroquine (*n* = 3). (E) Immunoblots for AR in C2C12‐AR97Q cells treated with 0.5‐mM AICAR for 0 (control) or 24 h and incubated with 1‐mg/mL cycloheximide (CHX) for 0, 3 and 6 h (*n* = 3). (F) Immunoblots for AR in C2C12‐AR97Q cells treated with 0.5‐mM AICAR for 0 (control) and 24 h, AICAR with 1‐μM MG132 or AICAR with 100‐nM bafilomycin (Baf). Graphs show densitometry quantification of relative AR protein (*n* = 3). *P < 0.05 by (A, C, F) one‐way ANOVA with Tukey's post hoc tests, (D) unpaired *t* test or (E) a two‐way ANOVA with Bonferroni post hoc test.

Finally, we investigated the effect of AMPK activation on AR aggregation in C2C12 muscle cells stably expressing *AR* with 97 CAG repeats (C2C12‐AR97Q). We used a well‐established exercise mimetic AICAR to activate AMPK pathway^S17^ in the C2C12‐AR97Q cells. AICAR at 0.5 mM successfully phosphorylated Thr‐172 of AMPK (*Figure* *6* *A*) and induced mitochondrial biogenesis (*Figure* *6* *B*) as observed in skeletal muscles of AR97Q mice in the early exercise group. Furthermore, AICAR treatment decreased phosphorylation levels of S6K1 (~75% reduction at 24 h, *P* < 0.001) and 4EBP1 (~20% reduction at 24 h, *P* < 0.001) and reduced AR97Q protein levels (~30% reduction at 24 h, *P* < 0.05) without inhibiting mRNA expression of mutant *AR* transgene (*Figure* *6* *C*). No change was observed in the protein levels of AR‐related molecular chaperones,^35^ heat shock protein 70 (Hsp70) and heat shock protein 40 (Hsp40) (*Figure* *6* *C*). Immunoblots for AR in non‐reducing conditions showed that AICAR treatment reduced both AR complex (~45% reduction at 24 h, *P* < 0.001) and monomer (~30% reduction at 24 h, *P* < 0.05) levels (*Figure* *S7A*). However, AICAR treatment did not reduce WT AR protein in C2C12 cells stably expressing *AR* with 24 CAG repeats (C2C12‐AR24Q), although it phosphorylated AMPK and decreased phosphorylation levels of S6K1 and 4EBP1 (*Figure* *S7B*). To investigate the mechanism of AICAR treatment of reducing AR97Q protein in the C2C12 cells, we assessed autophagy activity and protein degradation rate. Autophagy activity was estimated by measuring changes in LC3‐II levels to glyceraldehyde 3‐phosphate dehydrogenase (GAPDH). When we analysed LC3‐II levels after treatment of an autophagy inhibitor chloroquine, we observed reduced LC3‐II flux under AICAR treatment (*Figure* *6* *D*). AR97Q protein degradation rate was analysed by treating the C2C12‐AR97Q cells with CHX, which inhibits translation. The CHX chase assay showed that AICAR treatment enhanced AR97Q protein degradation (*Figure* *6* *E*). Furthermore, we treated the C2C12‐AR97Q cells with a proteasome inhibitor, MG132, or a lysosome inhibitor, bafilomycin A1, to clarify which pathway was responsible for AR degradation by the AICAR treatment. When the C2C12‐AR97Q cells were treated with MG132, AICAR‐induced reduction of AR proteins was counteracted, despite increased phosphorylation levels of AMPK (*Figure* *6* *F*). In contrast, treatment with bafilomycin A1 did not affect the reduction of AR proteins by AICAR (*Figure* *6* *F*). These results suggest that AMPK activation inhibited protein synthesis and reduced polyQ‐expanded AR aggregation through enhancement of protein degradation in proteasome.

## Discussion

The present study showed potential benefits of early physical exercise in SBMA. We demonstrated that aerobic exercise during the early stage improved behavioural and histopathological characteristics of AR97Q mice and extended their life span. In addition, the study finding also showed that this improvement was associated with exercise‐induced AMPK signalling activation in skeletal muscles.

We found that the exercise reduced polyQ‐expanded AR accumulation and ameliorated both neuronal pathology and muscular pathology in AR97Q mice. In the skeletal muscles, this effect was observed at 9 weeks of age, the end of exercise period, and sustained until 13 weeks of age, the end stage of nontreated mice. In the spinal cord, AR accumulation was almost at the same level to that in the sedentary mice at the end of exercise, but it decreased later. These observations implied that the amelioration of motor neuron pathology was not directly mediated by the exercise. Given that previous studies have shown that muscle pathology precedes motor neuron pathology, and altered trophic support from skeletal muscle may be responsible for non‐cell autonomous damage of motor neuron in SBMA,^8^, ^10^ skeletal muscle improvement by the early aerobic exercise might have ameliorated later motor neuron pathology in a non‐cell autonomous manner. A similar phenomenon to this study was previously reported, which showed that muscle‐restricted overexpression of IGF‐1 attenuated AR aggregation in both the skeletal muscles and the spinal cord of AR97Q mice.^9^ These findings and the results of the present study indicated that therapies targeting skeletal muscles do not only attenuate muscular degeneration but also have the potential to ameliorate motor neuron pathology in SBMA.

Microarray and biochemical analyses suggest that the reduction of polyQ‐expanded AR is associated with exercise‐induced AMPK signalling activation in skeletal muscles of AR97Q mice. We found that the exercise increased Thr‐172 phosphorylation levels of AMPK and induced expression of mitochondrial complex genes, which are well‐established muscle responses to physical exercise.^S9^ Although the low‐ to moderate‐intensity exercise used in this study had only modest effects in WT mice, it might have been a sufficient load to activate AMPK signalling in skeletal muscle of AR97Q mice. Indeed, the expression of *CS*, a muscle indicator of adaptation to exercise, was elevated by the exercise in AR97Q mice but not in WT mice. We also found that the exercise inhibited mTOR pathway that regulates protein synthesis. Interestingly, previous studies have shown that activation of mTOR signalling is an early feature in SBMA muscles^31^ and that AMPK pathway can inhibit mTOR signalling in skeletal muscles.^S18^ ^,^ ^S19^ Taken together, our results suggest that exercise‐induced AMPK activation counteracted the mTOR signalling and deactivated the abnormal protein synthesis in skeletal muscles of AR97Q mice.

We also demonstrated that AICAR‐mediated AMPK activation inhibited protein synthesis, facilitated polyQ‐expanded AR degradation and reduced AR aggregation in C2C12 cells. In contrast, AICAR treatment did not reduced WT AR, suggesting that AMPK activation has different effects on WT and aggregation‐prone polyQ proteins. Pharmacological activation of AMPK was reported to attenuate polyQ protein aggregation in mouse models of Machado–Joseph disease and Huntington's disease.^36^, ^37^ These studies demonstrated that activation of AMPK induced autophagy, which resulted in reduction of abnormal protein aggregation. However, the decrease of polyQ‐expanded AR by AICAR was unlikely to be mediated by autophagy in the present study. In the C2C12 cells, AICAR suppressed LC3‐II flux, which indicated that autophagy pathway was inhibited by the treatment. Correspondingly, inhibition of autophagy by bafilomycin A1 did not affect the reduction of AR protein by AICAR. Given that inhibition of proteasome by MG132 counteracted the AR reduction, proteasomal degradation might be responsible for the decrease in AR protein upon AMPK activation. Interestingly, a previous study showed that an mTOR inhibitor, rapamycin, inhibits polyQ protein aggregation by reducing protein synthesis independent of autophagy.^38^ A reduction in global protein synthesis might result in a decrease in the concentration of oligomers required for protein aggregation, which facilitates the degradation of polyQ proteins in proteasome. As aberrant autophagic response may exacerbate skeletal muscle atrophy and worsen the disease phenotype in SBMA,^39^, ^40^ modulating protein synthesis pathway by AMPK activation can be an attractive therapeutic strategy to reduce AR aggregation without inducing autophagy.

The optimal population of SBMA patients who would benefit from exercise remains to be clarified. Although the present results reveal that early aerobic exercise is effective on polyQ‐mediated neuromuscular degeneration, some cohorts of AR97Q mice at advanced stages were unable to complete the aerobic exercise, suggesting that tolerance to exercise varies with disease status in SBMA. Identifying objective indicators of exercise tolerance, such as specific biomarkers or clinical scores, may be necessary for implementing exercise therapy. It may also be necessary to adjust the exercise duration and intensity for patients with reduced exercise tolerance. In addition, housing condition of mice also should be considered. As physical inactivity can decrease motor performance,^S20^ we should take into account the possibility that the sedentary condition may affect the disease progression of AR97Q mice, and the intensity of aerobic exercise used in this study might correspond to the activity of daily living in patients with SBMA.

In conclusion, the present study showed that aerobic exercise during the early stage reduced polyQ‐expanded AR accumulation with amelioration of motor dysfunction in AR97Q mice. The reduction of AR aggregation was associated with exercise‐induced AMPK signalling activation in skeletal muscles, which improved both skeletal muscle pathology and spinal cord pathology. Physical exercise and AMPK activation may be effective in SBMA and other neuromuscular disorders caused by abnormal protein accumulation in skeletal muscles.

## Conflict of interest statement

The authors declare no competing interests.

## Funding

This work was funded in part by the Japan Society for the Promotion of Science (JSPS) KAKENHI (Grant Numbers JP20H00527, JP22K15706, and JP23H00420) and Japan Agency for Medical Research and Development (AMED) (Grant Numbers JP22nk0101575, JP22am0401007, and JP23bm1423003).

## Supporting information


**Figure S1.** Study design and exercise intensity. (A) Aerobic exercise (5 m/min) was performed from 5 to 9 weeks of age in wild‐type (WT) and AR97Q mice, and the phenotype of the mice was analyzed throughout the entire period. (B) Expression levels of *citrate synthas*e (*CS*) in the quadriceps of WT and AR97Q mice after 4 weeks of 5 m/min exercise.
**Figure S2.** Effect of exercise on behavior of wild‐type mice. Exercise was started from 5 weeks of age and maintained for 4 weeks in wild‐type (WT) mice. (A) Body weight, (B) grip strength, (C) rotarod performance, and (D) survival rate of WT mice in the sedentary (Sed, *n* = 14) and exercise (Ex, *n* = 15) groups are shown. Error bars indicate the standard error of the mean.
**Figure S3.** Analysis of microarray data from the quadriceps of AR97Q mice at 9 weeks of age between the sedentary and exercise groups. (A) Principal component analysis. (B) GO term enrichment analysis of differentially expressed genes (DEGs) between the sedentary and early exercise groups (*n* = 3 per group). Top five GO terms (Cellular Component) of DEGs (false discovery rate < 0.05, fold‐change ≤ 0.5 or ≥ 2) are listed. All of the top five categories are upregulated in the exercise group. The underlined genes are specifically expressed in fast‐twitch skeletal muscles. Sed, sedentary; Ex, exercise.
**Figure S4.** Differentially expressed genes of microarray data from the quadriceps of AR97Q mice between the sedentary and exercise groups. The top 30 differentially expressed genes that are up‐ (A) or downregulated (B).
**Figure S5.** KEGG AMPK signaling pathway in the quadriceps of AR97Q mice. KEGG pathway analysis is performed by iDEP using the microarray data. Red and green represent up‐ and downregulated genes in the exercise group compared to the sedentary group, respectively. Red and blue circles indicate mitochondrial biogenesis and protein synthesis pathway, respectively.
**Figure S6.** Autophagy, Igf‐1/Akt signaling, and BDNF expression are not elevated by exercise in skeletal muscles of AR97Q. (A) Log fold‐change (FC) of expression levels (Ex/Sed) of HGNC (https://www.genenames.org/) autophagy‐related genes from microarray data of the quadriceps of AR97Q mice at 9 weeks of age. Red and blue represent up‐ and downregulated genes in the exercise group, respectively. (B) Immunoblots for Becn1 and LC3 of the quadriceps of WT and AR97Q mice (*n* = 3 per group). (C) Log FC (Ex/Sed) and false discovery rate (FDR) of *Igf1* and *Bdnf* expression levels from microarray data of the quadriceps of AR97Q mice. (D) Immunoblots for phosphorylated (Ser‐473) and total Akt of the quadriceps of AR97Q mice (*n* = 5 per group). Sed, sedentary; Ex, exercise, *P < 0.05 by unpaired t‐test.
**Figure S7.** AMPK activation by AICAR reduces polyQ‐expanded AR aggregation in C2C12 cells. (A) Immunoblots for AR in non‐reducing conditions in C2C12‐AR97Q cells treated with 0.5 mM AICAR for 0, 8 or 24 h. Graphs show densitometry quantification of AR complex and AR monomer levels (*n* = 3). (B) Immunoblots for AR, phosphorylation (Thr‐172), total AMPK, phosphorylation (Thr‐389), total S6K1, and 4EBP1 in C2C12‐AR24Q cells treated with 0.5 mM AICAR for 0 or 24 h. *P < 0.05 by one‐way ANOVA with Tukey's post hoc tests (A) or unpaired t‐test (B).

**Data S1.** Supporting Information

**Data S2.** Supporting Information
