# Supplementary material for: Exercise attenuates polyglutamine‐mediated neuromuscular degeneration in a mouse model of spinal and bulbar muscular atrophy
Source: J Cachexia Sarcopenia Muscle. 2023 Nov 8;15(1):159–72. doi: 10.1002/jcsm.13344 (PMC10834330; doi:10.1002/jcsm.13344)
Supplement: Supplementary file 1 — Figure S1. Study design and exercise intensity. (A) Aerobic exercise (5 m/min) was performed from 5 to 9 weeks of age in wild‐type (WT) and AR97Q mice, and the phenotype of the mice was analyzed throughout the entire period. (B) Expression levels of citrate synthase (CS) in the quadriceps of WT and AR97Q mice after 4 weeks of 5 m/min exercise. Figure S2. Effect of exercise on behavior of wild‐type mice. Exercise was started from 5 weeks of age and maintained for 4 weeks in wild‐type (WT) mice. (A) Body weight, (B) grip strength, (C) rotarod performance, and (D) survival rate of WT mice in the sedentary (Sed, n = 14) and exercise (Ex, n = 15) groups are shown. Error bars indicate the standard error of the mean. Figure S3. Analysis of microarray data from the quadriceps of AR97Q mice at 9 weeks of age between the sedentary and exercise groups. (A) Principal component analysis. (B) GO term enrichment analysis of differentially expressed genes (DEGs) between the sedentary and early exercise groups (n = 3 per group). Top five GO terms (Cellular Component) of DEGs (false discovery rate < 0.05, fold‐change ≤ 0.5 or ≥ 2) are listed. All of the top five categories are upregulated in the exercise group. The underlined genes are specifically expressed in fast‐twitch skeletal muscles. Sed, sedentary; Ex, exercise. Figure S4. Differentially expressed genes of microarray data from the quadriceps of AR97Q mice between the sedentary and exercise groups. The top 30 differentially expressed genes that are up‐ (A) or downregulated (B). Figure S5. KEGG AMPK signaling pathway in the quadriceps of AR97Q mice. KEGG pathway analysis is performed by iDEP using the microarray data. Red and green represent up‐ and downregulated genes in the exercise group compared to the sedentary group, respectively. Red and blue circles indicate mitochondrial biogenesis and protein synthesis pathway, respectively. Figure S6. Autophagy, Igf‐1/Akt signaling, and BDNF expression are not elevated by exercise in [file JCSM-15-159-s002.pdf]

Exercise attenuates polyglutamine-mediated neuromuscular degeneration in a mouse model of spinal and bulbar muscular atrophy

Tomoki Hirunagi, Hideaki Nakatsuji, Kentaro Sahashi, Mikiyasu Yamamoto, Madoka Iida, Genki Tohnai, Naohide Kondo, Shinichiro Yamada, Ayuka Murakami, Seiya Noda, Hiroaki Adachi, Gen Sobue, and Masahisa Katsuno

SUPPLEMENTAL FIGURES

A

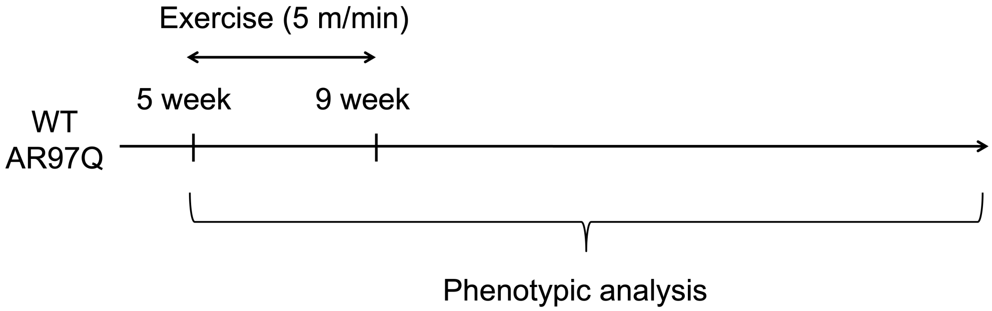

B

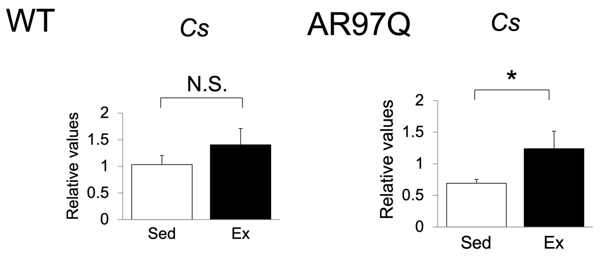

Figure S1 Study design and exercise intensity. (**A**) Aerobic exercise (5 m/min) was performed from 5 to 9 weeks of age in wild-type (WT) and AR97Q mice, and the phenotype of the mice was analyzed throughout the entire period. (**B**) Expression levels of *citrate synthas*e (*CS*) in the quadriceps of WT and AR97Q mice after 4 weeks of 5 m/min exercise.

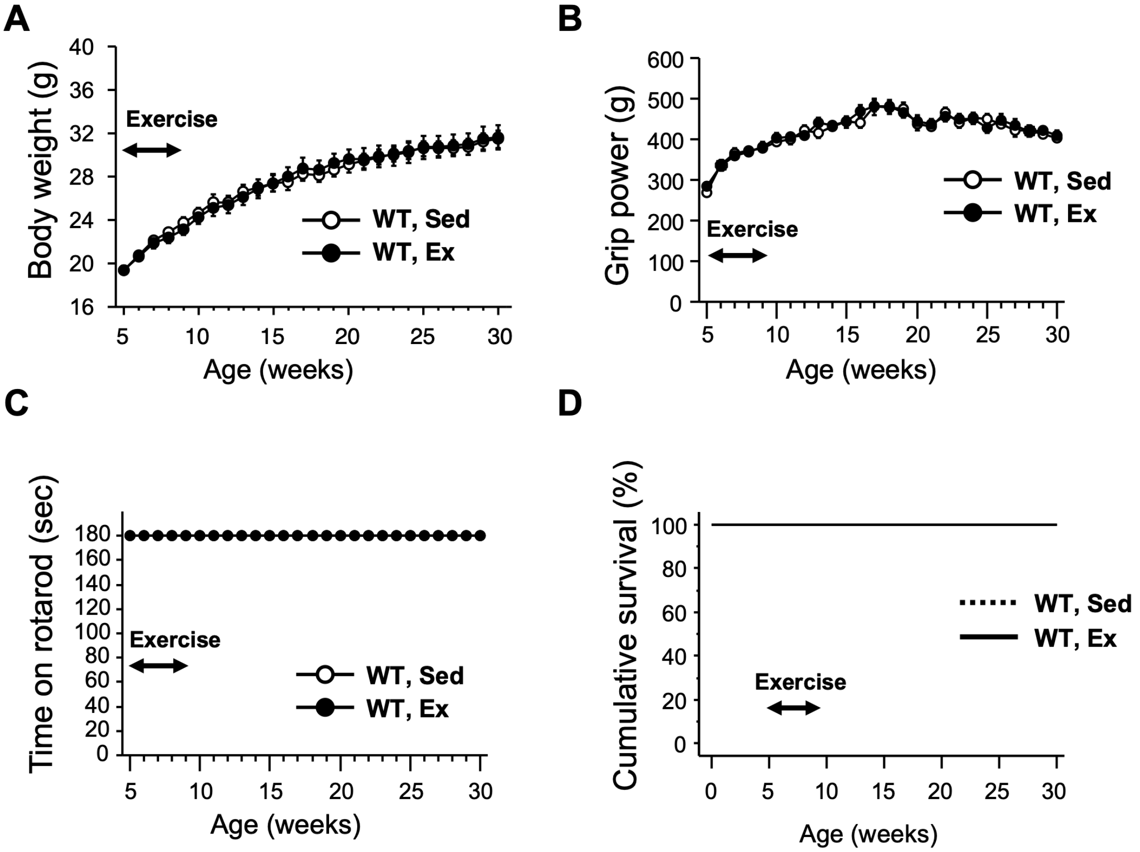

Figure S2 Effect of exercise on behavior of wild-type mice. Exercise was started from 5 weeks of age and maintained for 4 weeks in wild-type (WT) mice. (**A**) Body weight, (**B**) grip strength, (**C**) rotarod performance, and (**D**) survival rate of WT mice in the sedentary (Sed, n = 14) and exercise (Ex, n = 15) groups are shown. Error bars indicate the standard error of the mean.

A

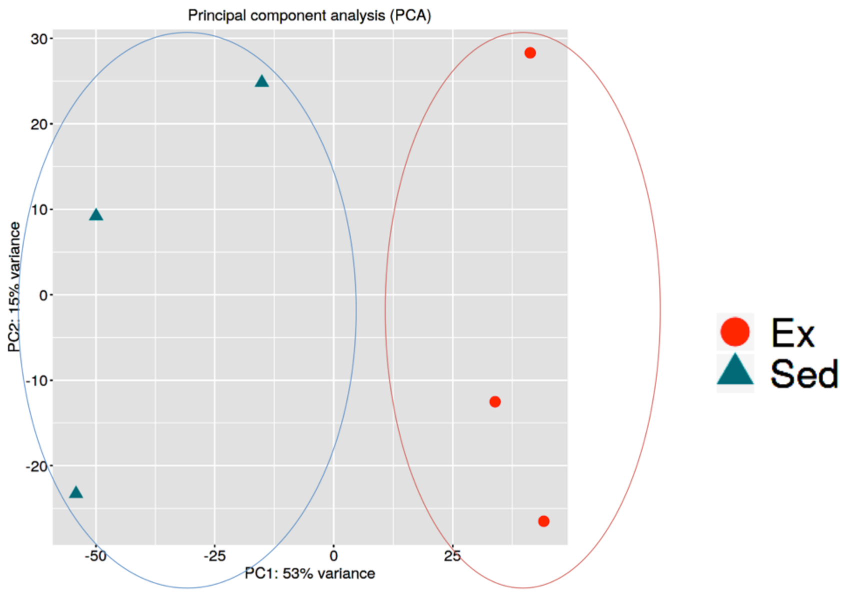

B

	Direction	Pathway	Genes
1	Up regulated	Sarcoplasmic reticulum membrane	Atp2a1 Dhrs7c Casq1 Jph2 Art1
2	Up regulated	Contractile fiber	Tmod4 Casq1 Mybpc2 Myoz1 Actn3 Jph2 Acta2 Npnt Myl1 Atp2a1
3	Up regulated	Contractile fiber part	Tmod4 Casq1 Mybpc2 Myoz1 Actn3 Jph2 Acta2 Npnt Atp2a1
4	Up regulated	Sarcoplasmic reticulum	Atp2a1 Dhrs7c Casq1 Jph2 Art1
5	Up regulated	Myofibril	Tmod4 Casq1 Mybpc2 Myoz1 Actn3 Jph2 Myl1 Atp2a1

Figure S3 Analysis of microarray data from the quadriceps of AR97Q mice at 9 weeks of age between the sedentary and exercise groups. (**A**) Principal component analysis. (**B**) GO term enrichment analysis of differentially expressed genes (DEGs) between the sedentary and early exercise groups (n = 3 per group). Top five GO terms (Cellular Component) of DEGs (false discovery rate < 0.05, fold-change ≤ 0.5 or ≥ 2) are listed. All of the top five categories are upregulated in the exercise group. The underlined genes are specifically expressed in fast-twitch skeletal muscles. Sed, sedentary; Ex, exercise.

A

Symbol	log2 FC	Adj.Pval	Ensembl ID
1700001O22Rik	3.288489	2.35E-02	ENSMUSG00000044320
Ppm1n	2.831215	2.13E-02	ENSMUSG00000030402
Smox	2.562077	2.60E-02	ENSMUSG00000027333
2310065F04Rik	2.506819	2.38E-02	ENSMUSG00000087410
Cacna2d4	2.342422	2.61E-02	ENSMUSG00000041460
Nmrk2	2.320873	2.61E-02	ENSMUSG00000004939
Cldn3	2.252005	2.61E-02	ENSMUSG00000070473
Dmkn	2.171951	2.61E-02	ENSMUSG00000060962
Kcnc1	2.159699	3.19E-02	ENSMUSG00000058975
BC049352	2.157911	2.61E-02	ENSMUSG00000091996
Kcng4	2.095916	2.35E-02	ENSMUSG00000045246
Dnase1	2.075303	2.88E-02	ENSMUSG00000005980
Nppc	2.040938	2.61E-02	ENSMUSG00000026241
7420701I03Rik	2.021526	2.42E-02	ENSMUSG00000113620
Kcnf1	1.997373	3.04E-02	ENSMUSG00000051726
Cuzd1	1.986606	4.84E-02	ENSMUSG00000040205
A230009B12Rik	1.966165	2.13E-02	ENSMUSG00000089633
Cited4	1.959699	2.46E-02	ENSMUSG00000070803
Tbc1d10c	1.91046	2.13E-02	ENSMUSG00000040247
Mettl21c	1.895102	2.49E-02	ENSMUSG00000047343
Fosb	1.893266	3.63E-02	ENSMUSG00000003545
Cxcl1	1.873316	2.35E-02	ENSMUSG00000029380
Hsd17b13	1.86441	4.03E-02	ENSMUSG00000034528
Amd2	1.846546	2.61E-02	ENSMUSG00000063953
Fos	1.831821	3.24E-02	ENSMUSG00000021250
4933401D09Rik	1.822648	2.38E-02	ENSMUSG00000116924
Mfsd4b5	1.796745	3.66E-02	ENSMUSG00000038528
Adam7	1.786005	4.06E-02	ENSMUSG00000022056
Smco1	1.783159	3.78E-02	ENSMUSG00000046345
Vmn1r82	1.75034	3.61E-02	ENSMUSG00000058132

B

Symbol	log2 FC	Adj.Pval	Ensembl ID
Chrng	-3.76703	3.38E-02	ENSMUSG00000026253
Plekhh1	-3.65932	2.44E-02	ENSMUSG00000060716
AI606473	-3.07208	3.38E-02	ENSMUSG00000093738
Slc18a1	-2.83115	2.96E-02	ENSMUSG00000036330
Lhx1os	-2.83097	3.55E-02	ENSMUSG00000087211
Pax6	-2.78246	3.14E-02	ENSMUSG00000027168
Tfap2a	-2.57868	2.85E-02	ENSMUSG00000021359
Myh3	-2.50069	4.06E-02	ENSMUSG00000020908
Scn5a	-2.49491	2.87E-02	ENSMUSG00000032511
Foxl2os	-2.44478	4.79E-02	ENSMUSG00000097072
Prr32	-2.43879	2.80E-02	ENSMUSG00000037086
Tmem181b-ps	-2.40967	2.87E-02	ENSMUSG00000096780
Isl1	-2.38564	3.55E-02	ENSMUSG00000042258
Arhgap36	-2.37811	2.42E-02	ENSMUSG00000036198
Stum	-2.37084	2.61E-02	ENSMUSG00000053963
Dlg2	-2.29985	2.87E-02	ENSMUSG00000052572
Nefh	-2.28634	2.61E-02	ENSMUSG00000020396
Col19a1	-2.27768	3.19E-02	ENSMUSG00000026141
Lhx8	-2.24755	3.46E-02	ENSMUSG00000096225
Usp29	-2.23554	2.19E-02	ENSMUSG00000051527
Lhx1	-2.22002	4.55E-02	ENSMUSG00000018698
Gata4	-2.19872	4.75E-02	ENSMUSG00000021944
Slc2a5	-2.15756	2.98E-02	ENSMUSG00000028976
Ano3	-2.12101	3.05E-02	ENSMUSG00000074968
Pnpla3	-2.07781	4.94E-02	ENSMUSG00000041653
Chrnd	-2.07757	3.61E-02	ENSMUSG00000026251
Klra7	-2.0773	3.49E-02	ENSMUSG00000067599
Sln	-2.07304	3.95E-02	ENSMUSG00000042045
Chgb	-2.05863	2.35E-02	ENSMUSG00000027350
Prss16	-2.05144	2.85E-02	ENSMUSG00000006179

Figure S4 Differentially expressed genes of microarray data from the quadriceps of AR97Q mice between the sedentary and exercise groups. The top 30 differentially expressed genes that are up- (**A**) or downregulated (**B**).

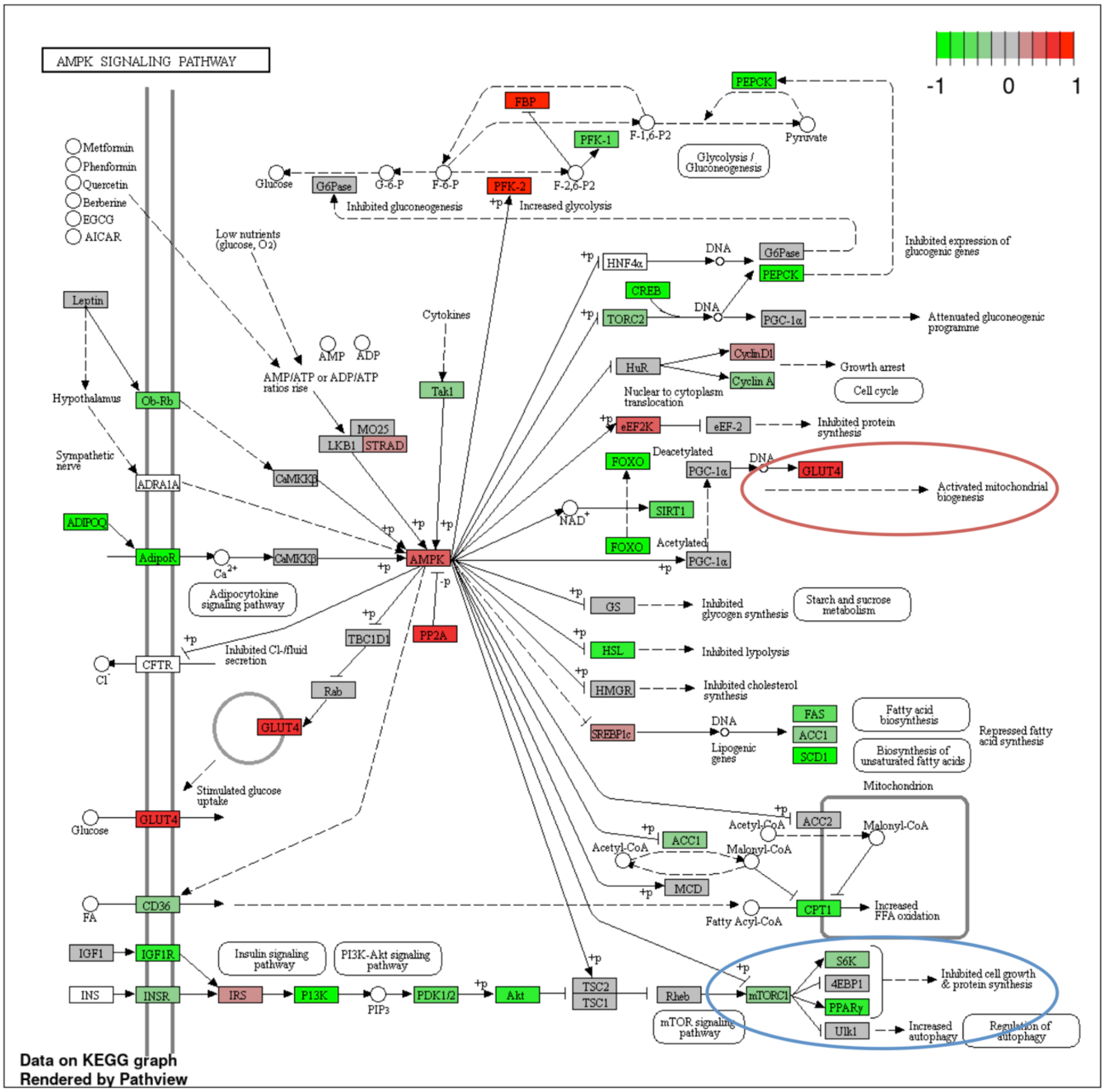

Figure S5 KEGG AMPK signaling pathway in the quadriceps of AR97Q mice. KEGG pathway analysis is performed by iDEP using the microarray data. Red and green represent up- and downregulated genes in the exercise group compared to the sedentary group, respectively. Red and blue circles indicate mitochondrial biogenesis and protein synthesis pathway, respectively.

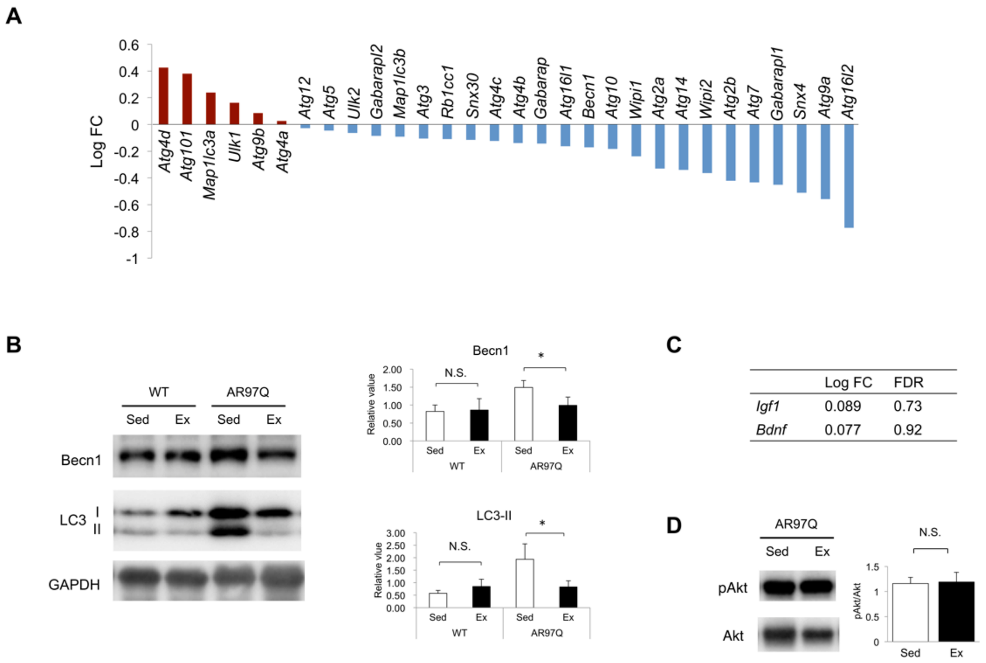

Figure S6 Autophagy, Igf-1/Akt signaling, and BDNF expression are not elevated by exercise in skeletal muscles of AR97Q. (**A**) Log fold-change (FC) of expression levels (Ex/Sed) of HGNC (https://www.genenames.org/) autophagy-related genes from microarray data of the quadriceps of AR97Q mice at 9 weeks of age. Red and blue represent up- and downregulated genes in the exercise group, respectively. (**B**) Immunoblots for Becn1 and LC3 of the quadriceps of WT and AR97Q mice (n = 3 per group). (**C**) Log FC (Ex/Sed) and false discovery rate (FDR) of *Igf1* and *Bdnf* expression levels from microarray data of the quadriceps of AR97Q mice. (**D**) Immunoblots for phosphorylated (Ser-473) and total Akt of the quadriceps of AR97Q mice (n = 5 per group). Sed, sedentary; Ex, exercise, *$P < 0.05$ by unpaired t-test.

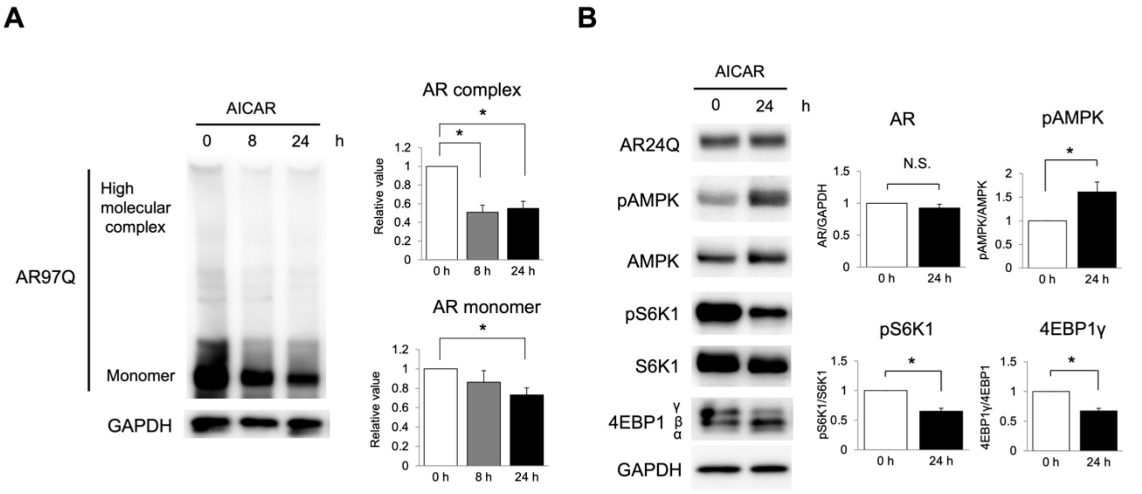

Figure S7 AMPK activation by AICAR reduces polyQ-expanded AR aggregation in C2C12 cells. (**A**) Immunoblots for AR in non-reducing conditions in C2C12-AR97Q cells treated with 0.5 mM AICAR for 0, 8 or 24 h. Graphs show densitometry quantification of AR complex and AR monomer levels (n = 3). (**B**) Immunoblots for AR, phosphorylation (Thr-172), total AMPK, phosphorylation (Thr-389), total S6K1, and 4EBP1 in C2C12-AR24Q cells treated with 0.5 mM AICAR for 0 or 24 h. *P < 0.05 by one-way ANOVA with Tukey's post hoc tests (**A**) or unpaired t-test (**B**).
