# Supplementary material for: Exercise attenuates polyglutamine‐mediated neuromuscular degeneration in a mouse model of spinal and bulbar muscular atrophy
Source: J Cachexia Sarcopenia Muscle. 2023 Nov 8;15(1):159–72. doi: 10.1002/jcsm.13344 (PMC10834330; doi:10.1002/jcsm.13344)
Supplement: Supplementary file 2 — Data S1. Supporting Information [file JCSM-15-159-s001.docx]

**Supplemental Methods**

**Immunoblotting**

Mouse tissues and cell lysates were homogenized in CelLytic lysis buffer (Sigma-Aldrich, St. Louis, MO) containing Halt Protease and Phosphatase Inhibitor Cocktails (Thermo Scientific, Waltham, MA). Homogenates were centrifuged at 2,500 g for 20 minutes at 4°C, and the supernatants were boiled in Sample Buffer Solution (Fujifilm) with 10% 2-mercaptoethanol for 10 minutes. Equal amounts of protein were separated on 5%–20% or 15% SDS–PAGE gels and transferred to Hybond-P membranes (GE Healthcare, Piscataway, NJ). Immunoblot analysis was performed with the following primary antibodies: AR (1:2000, H280, Santa Cruz Biotechnology, Santa Cruz, CA); choline acetyltransferase (ChAT) (1:2000, AB144P, Millipore, Billerica, MA); beclin1 (Becn1) (1:2000, #3495, Cell Signaling Technology, Beverly, MA); microtubule-associated protein light-chain 3 (LC3) (1:2000, #12741, Cell Signaling Technology); AMPKα (1:2000, #2532S, Cell Signaling Technology); phospho-AMPKα (Thr-172) (1:1000, #2535S, Cell Signaling Technology); ribosomal protein S6 kinase B1 (S6K1) (1:2000, #9202S, Cell Signaling Technology); phospho-S6K1 (Thr 389) (1:1000, #9205, Cell Signaling Technology); eukaryotic translation initiation factor 4E binding protein 1 (4EBP1) (1:1000, #9452, Cell Signaling Technology); heat shock protein 70 (Hsp70) (1:2000, #4872, Cell Signaling Technology); Hsp40 (1:2000, #4868, Cell Signaling Technology) or glyceraldehyde 3-phosphate dehydrogenase (GAPDH) (1:5000, Cell Signaling Technology, #2118). This step was followed by incubation with secondary antibody conjugated with horseradish peroxidase at a 1:3000 dilution. The ECL Plus system (GE Healthcare) was used for detection of proteins. An LAS-3000 imaging system (Fujifilm, Tokyo, Japan) was used to produce digital images. The signal intensities of the blots were quantified using IMAGE GAUGE software version 4.22 (Fujifilm) or ImageJ software (NIH, Bethesda, MD) and expressed in arbitrary units.

**Histology and immunohistochemistry**

Tissues were dissected, post-fixed in 10% phosphate-buffered formalin, and processed for paraffin embedding. The sections to be stained with the anti-polyglutamine antibody (1C2; 1:20,000, MAB1574, Millipore) were treated with formic acid for 5 minutes at room temperature. The sections to be incubated with the anti-ChAT antibodies (1:2000, AB144P, Millipore) were boiled in 10 mM citrate buffer for 15 minutes. Primary antibody binding was probed using a secondary antibody labeled with a polymer as part of the Envision+ system using horseradish peroxidase (Dako Cytomation, Gostrup, Denmark). The immunohistochemical sections were photographed with an optical microscope (Axio Imager M1, Carl Zeiss AG, Go¨ttingen, Germany). To measure muscle fiber size, paraffin-embedded sections (6 mm thick) of the quadriceps muscles were air-dried and stained with hematoxylin and eosin. For preparation of frozen sections, quadriceps muscles were snap frozen in acetone. Sections of unfixed muscle tissue were cut at 10 μm in a cryostat and processed for nicotinamide adenine dinucleotide (NADH) and myosin heavy chain (MHC) staining. For NADH staining, slides were incubated in NADH and NBT solution at 37°C for 1 hour and placed in 10% formalin for 20 minutes. Slides were then rinsed and mounted with mounting medium. For MHC staining, cryostat sections were fixed in acetone for 10 minutes and stained with primary antibodies against MHC type I (1:50, BA-F8), type IIa (1:600, SC-71) and type IIb (1:100, BF-F3) (Developmental Studies Hybridoma Bank, Iowa, IA) in accordance with previous reports ^32,S21^. Immunoreactivity was detected using the following secondary antibodies: Alexa Fluor 647 IgG2b (1:500), Alexa Fluor 488 IgG1 (1:500), and Alexa Fluor 555 IgM (1:500) (Invitrogen, Carlsbad, CA). A fluorescent microscope (BZ-X710, Keyence, Osaka, Japan) was used to visualize slides.

**Quantitative analysis of immunohistochemistry**

To quantitatively assess the 1C2-positive cells, at least 50 consecutive 6-mm-thick axial sections of the thoracic spinal cord and skeletal muscle were prepared, and every fifth section was immunostained with each antibody. The numbers of 1C2-positive cells were counted in all the neurons of the anterior horn of the 10 axial sections from the thoracic spinal cord of each group of mice under a light microscope (Bx51; Olympus, Tokyo, Japan). Motor neurons were defined by their presence within the anterior horn and the obvious nucleolus in a specific 6-μm-thick section. The numbers of 1C2-positive cells in the quadriceps muscles were calculated for 500 fibers in randomly selected areas of the 10 axial sections. Muscle fiber and motor neuron sizes were measured by ImageJ software.

**Quantification of mRNA levels**

Total RNA was isolated from cell pellets and tissues homogenized in TRIzol Reagent (Thermo Scientific) using a PureLink Genomic DNA Mini Kit (Thermo Scientific) according to the protocol supplied by the manufacturer. Total RNA was reverse transcribed using SuperScript III Reverse Transcriptase (Thermo Scientific). Real-time quantitative PCR amplification was performed using the iCycler system (Bio-Rad, Hercules, CA) using the Thunderbird qPCR mix (Toyobo, Osaka, Japan) according to the protocol recommended by the manufacturer. The results for each sample were normalized to the respective *beta-2 microglobulin* (*B2m*) mRNA levels. The primer sets were as follows: *B2m*, 5′-CTGACCGGCCTGTATGCTAT-3′ (forward) and 5′-CCGTTCTTCAGCATTTGGAT-3′ (reverse); *Cs*, 5’-GGACAATTTTCCAACCAATCTGC-3’ (forward) and 5’-TCGGTTCATTCCCTCTGCATA-3’ (reverse); human *AR*, 5′-CGGAAGCTGAAGAAACTTGG-3′ (forward) and 5′-ATGGCTTCCAGGACATTCAG-3′ (reverse); *Atp2a1*, 5′-TGTTTGTCCTATTTCGGGGTG-3′(forward) and 5′-AATCCGCACAAGCAGGTCTTC-3′ (reverse); *Casq1*, 5′-ATGAGAGCTACCGACAGGATG-3′ (forward) and 5′-CACCGTCGTACTCAGGGAAG-3′ (reverse); *Jph2*, 5′-CCAAGGGCCAGGGTGAATAC-3′ (forward) and 5′-TGGCTCCAATATCCCTCAAAGG-3′ (reverse); *Dhrs7c*, 5′-AGGTCCTGGACTGCTACGG-3′ (forward) and 5′-GGGTCCGAAGTAGTTGGCATC-3′ (reverse); *Art1* 5′-ATTCCTGCTATGATGTCTCTCCT-3′ (forward) and 5′-TGTCCAGGGGTGTTTCTTGAG-3′ (reverse).

**Microarray analysis**

Male AR97Q mice were examined at 9 weeks of age for each group (sedentary or exercise, n = 3 per group). Total RNA was isolated as described above. The quality of total RNA was assessed with Agilent 2200 TapeStation, and and the RNA integrity number values showed sufficiently high quality. The cDNA preparation, hybridization process and microarray data analysis were performed by TAKARA BIO (Otsu, Japan). Slides were scanned immediately after washing on the Agilent SureScan Microarray Scanner (G2600D) using one color scan setting for 8×60k array slides (scan area 61×21.6 mm, scan resolution 3 μm, dye channel was set to Green, and PMT was set to 100%). The scanned images were analyzed with Feature Extraction Software (Agilent Technologies) using default parameters to obtain background subtracted and spatially detrended Processed Signal intensities. Expression data were analyzed using iDEP (http://bioinformatics.sdstate.edu/idep90/) ^S22^. Genes with false discovery rate (FDR) < 0.05 and absolute Log fold-change (FC) ≥ 1 were defined as differentially expressed genes (DEGs) between each group. Parametric Gene Set Enrichment Analysis (PGSEA) was performed to identify sets of related genes altered in each group.
