# Supplementary material for: Exercise attenuates polyglutamine‐mediated neuromuscular degeneration in a mouse model of spinal and bulbar muscular atrophy
Source: J Cachexia Sarcopenia Muscle. 2023 Nov 8;15(1):159–72. doi: 10.1002/jcsm.13344 (PMC10834330; doi:10.1002/jcsm.13344)
Supplement: Supplementary file 3 — Data S2. Supporting Information [file JCSM-15-159-s003.docx]

S1. Liu Y, Yan T, Chu JMT, Chen Y, Dunnett S, Ho YS *et al.* The beneficial effects of physical exercise in the brain and related pathophysiological mechanisms in neurodegenerative diseases. *Lab Investig* 2019;**99**:943–957.

S2. Fernando P, Bonen A, Hoffman-Goetz L. Predicting submaximal oxygen consumption during treadmill running in mice. *Can J Physiol Pharmacol***71**:854–857.

S3. Siu PM, Donley DA, Bryner RW, Alway SE. Citrate synthase expression and enzyme activity after endurance training in cardiac and skeletal muscles. *J Appl Physiol* 2003;**94**:555–560.

S4. Kaneb HM, Sharp PS, Rahmani-Kondori N, Wells DJ. Metformin treatment has no beneficial effect in a dose-response survival study in the SOD1 G93A mouse model of ALS and is harmful in female mice. *PLoS One* 2011;**6**: e24189.

S5. Ringer C, Büning LS, Schäfer MKH, Eiden LE, Weihe E, Schütz B. PACAP signaling exerts opposing effects on neuroprotection and neuroinflammation during disease progression in the SOD1(G93A) mouse model of amyotrophic lateral sclerosis. *Neurobiol Dis* 2013;**54**:32–42.

S6. Kim SY, Volsky DJ. PAGE: Parametric analysis of gene set enrichment. *BMC Bioinformatics* 2005;**6**:1–12.

S7. Jørgensen SB, Richter EA, Wojtaszewski JFP. Role of AMPK in skeletal muscle metabolic regulation and adaptation in relation to exercise. *J Physiol* 2006;**574**:17–31.

S8. Brandauer J, Andersen MA, Kellezi H, Risis S, Frøsig C, Vienberg SG *et al.* AMP-activated protein kinase controls exercise training- and AICAR-induced increases in SIRT3 and MnSOD. *Front Physiol* 2015;**6**:1–16.

S9. Kanehisa M, Furumichi M, Sato Y, Ishiguro-Watanabe M, Tanabe M. KEGG: Integrating viruses and cellular organisms. *Nucleic Acids Res* 2021;**49**:D545–D551.

S10. Luo W, Brouwer C. Pathview: An R/Bioconductor package for pathway-based data integration and visualization. *Bioinformatics* 2013;**29**:1830–1831.

S11. Ojuka EO, Jones TE, Nolte LA, Chen M, Wamhoff BR, Sturek M *et al.* Regulation of GLUT4 biogenesis in muscle: Evidence for involvement of AMPK and Ca2+. *Am J Physiol - Endocrinol Metab* 2002;**282**:1008–1013.

S12. Shah OJ, Anthony JC, Kimball SR, Jefferson LS. 4E-BP1 and S6K1: translational integration sites for nutritional and hormonal information in muscle. *Am J Physiol Endocrinol Metab* 2000;**279**:E715-E729.

S13. Kim J, Kundu M, Viollet B, Guan KL. AMPK and mTOR regulate autophagy through direct phosphorylation of Ulk1. *Nat Cell Biol* 2011;**13**:132–141.

S14. Eskelinen EL, Saftig P. Autophagy: A lysosomal degradation pathway with a central role in health and disease. *Biochim Biophys Acta - Mol Cell Res* 2009;**1793**:664–673.

S15. Pedersen BK, Febbraio MA. Muscles, exercise and obesity: Skeletal muscle as a secretory organ. *Nat Rev Endocrinol* 2012;**8**:457–465.

S16. Kjøbsted R, Hingst JR, Fentz J, Foretz M, Sanz MN, Pehmøller C *et al.* AMPK in skeletal muscle function and metabolism. *FASEB J* 2018;**32**:1741–1777.

S17. Narkar VA, Downes M, Yu RT, Embler E, Wang YX, Banayo E *et al.* AMPK and PPARδ Agonists Are Exercise Mimetics. *Cell* 2008;**134**:405–415.

S18. Bolster DR, Crozier SJ, Kimball SR, Jefferson LS. AMP-activated protein kinase suppresses protein synthesis in rat skeletal muscle through down-regulated mammalian target of rapamycin (mTOR) signaling. *J Biol Chem* 2002;**277**:23977–23980.

S19. Drake JC, Alway SE, Hollander JM, Williamson DL. AICAR treatment for 14 days normalizes obesity-induced dysregulation of TORC1 signaling and translational capacity in fasted skeletal muscle. *Am J Physiol - Regul Integr Comp Physiol* 2010;**299**:1546–1554.

S20. Roemers P, Hulst Y, van Heijningen S, van Dijk G, van Heuvelen MJG, De Deyn PP *et al.* Inducing Physical Inactivity in Mice: Preventing Climbing and Reducing Cage Size Negatively Affect Physical Fitness and Body Composition. *Front Behav Neurosci* 2019;**13**:221.

S21. Bloemberg D, Quadrilatero J. Rapid determination of myosin heavy chain expression in rat, mouse, and human skeletal muscle using multicolor immunofluorescence analysis. *PLoS One* 2012;**7**: e35273.

S22. Ge SX, Son EW, Yao R. iDEP: An integrated web application for differential expression and pathway analysis of RNA-Seq data. *BMC Bioinformatics* 2018;**19**:1–24.
